# Comparison of the Grades of Fatty Liver Disease With Perioperative Risk Factors in Patients With Laparoscopic Sleeve Gastrectomy

**DOI:** 10.7759/cureus.69717

**Published:** 2024-09-19

**Authors:** Yalçın Burak Kara, Yahya Ozel

**Affiliations:** 1 Department of General Surgery, Bahcesehir University, School of Medicine, Istanbul, TUR; 2 Department of General Surgery, Medical Park Pendik Hospital, Istanbul, TUR; 3 General Surgery, Dogus University School of Medicine, Istanbul, TUR

**Keywords:** bariatric surgery, hepatosteatosis, laparoscopic sleeve gastrectomy, nonalcolic fatty liver disease, risk factors

## Abstract

Background

Obesity is a global healthcare problem, and nonalcoholic fatty liver disease (NAFLD) is a commonly observed comorbid disease in the bariatric population. This study evaluated the relationship between NAFLD and various risk factors, including demographic, biochemical, and comorbid conditions in patients undergoing laparoscopic sleeve gastrectomy (LSG).

Material and methods

This retrospective data analysis study included patients who underwent LSG between August 2023 and 2024. Patient demographic data were collected, such as age, gender, weight, and body mass index (BMI), and NAFLD grade was determined by ultrasonography. Biochemical markers were recorded, including alanine aminotransferase (ALT), aspartate aminotransferase (AST), total cholesterol, triglyceride (TG), high-density lipoprotein (HDL), low-density lipoprotein (LDL), fasting blood glucose (FBG), HbA1c, and vitamin D. The presence of type II diabetes mellitus (T2DM) and hypertension (HT) was evaluated and compared with the grade of hepatosteatosis.

Results

The study included 436 patients, of whom 73.6% (*n *= 321) were female. The mean age was 37.23 ± 10.49 years, and the mean BMI value was 41.25 ± 6.11 kg/m^2^. Patients were classified and compared according to their NAFLD grade, revealing statistically significant differences in weight, BMI, ALT, AST, HDL, LDL, TG, total cholesterol, HbA1c, FBG, vitamin D, obesity class, DM, and HT (*p < *0*.*05). HDL and vitamin D showed an inverse correlation with NAFLD. We observed no significant difference in the relationship of NAFLD with age and the presence of gallstone. Logistic regression analysis revealed that ALT, AST, LDL, total cholesterol, and FBG were statistically significantly associated with NAFLD in the multivariate model.

Conclusion

Hepatosteatosis, T2DM, and HT are frequent comorbid diseases that are common in bariatric patients. Our study shows that ALT, AST, LDL, FBG, and total cholesterol may be used as predictors of NAFLD.

## Introduction

The prevalence of obesity is increasing daily, with the condition expected to affect approximately two billion people by 2035. The vast number of individuals with obesity globally will lead to many obesity-related comorbid diseases requiring treatment, such as type 2 diabetes mellitus (T2DM), hypertension (HT), obstructive sleep apnea, dyslipidemia, nonalcoholic fatty liver disease (NAFLD), cardiovascular diseases (CVDs), bone and joint diseases, polycystic ovarian syndrome, and gastroesophageal reflux diseases [1-3]. Bariatric and metabolic surgeries are long-standing, efficacious treatment options for this population, and nearly 57.4% of all operations are laparoscopic sleeve gastrectomies (LSGs) [4].

NAFLD is a family of serious liver diseases ranging from simple hepatic steatosis without inflammation to nonalcoholic steatohepatitis (NASH) with serious inflammation and cellular damage, which is considered to cause liver cirrhosis [5,6]. NAFLD is more common in males, and its global incidence is believed to reach up to 32% [7]. The risk factors for NAFLD include insulin resistance, T2DM, HT, dyslipidemia, obesity, polycystic ovarian syndrome, and metabolic syndrome. Behavioral and environmental factors are also implicated in the development of NAFLD, especially a diet high in carbohydrate and fat. In addition, genetic risk factors, such as a mutation in patatin-like phospholipase, have been shown to be strongly relevant to NAFLD severity. Literature reviews have shown that vitamin E supplementation is beneficial for hepatic inflammation and that vitamin D deficiency is closely related to NAFLD and increases hepatic inflammation [5,6,8,9].

Steatohepatitis may cause hepatic inflammation and increase hepatic enzymes, such as alanine aminotransferase (ALT) and aspartate aminotransferase (AST). Studies show that increased adiposity, elevated body mass index (BMI), and elevated ALT level have strong relationships with NAFLD [10]. Furthermore, there is a complex interaction between NAFLD and T2DM. NAFLD has been shown to double the risk of T2DM, and central obesity and diabetes mellitus (DM) increase the risk of developing NAFLD [11,12]. CVDs are commonly observed in patients with NAFLD, which trigger atherosclerotic dyslipidemia as identified by an elevated triglyceride (TG) level, decreased high-density lipoprotein (HDL), and elevated low-density lipoprotein (LDL) [13,14].

While liver biopsy offers the most accurate diagnosis of NAFLD, it is usually clinically evaluated by ultrasonography (USG) due to the invasive nature of biopsy [5,8], as USG has high specificity and sensitivity for hepatosteatosis (89% and 92%, respectively), making it preferable to biopsy [15]. This study evaluated the relationship between hepatic steatosis and perioperative risk factors in patients undergoing LSG in our clinic.

## Materials and methods

Patients

This retrospective observational study included patients who underwent LSG consecutively between August 2023 and August 2024. We collected the patients’ demographic data, including age, gender, weight, height, and BMI. Their hepatosteatosis grades were determined by USG. The presence of gallstones was also reported. Biochemical markers were analyzed, including lipid profile (LDL (60-130 mg/dl), HDL (40-80 mg/dl), total cholesterol (50-200 mg/dl), and TG (60-150 mg/dl)); diabetic markers (HbA1c (4-5, 7%) and fasting blood glucose (FBG) (70-100 mg/dl)); hepatic profile (hepatitis B antigen (0.001-0.99) and hepatitis C antibody (0-1)); liver function tests (ALT (0-55 U/L) and AST (5-34 U/L)); and vitamin D (30-45 ng/ml). Patients were classified and recorded for obesity as follows: BMI 30.0-34.9 kg/m^2^, class 1; BMI 35.0-39.9 kg/m^2^, class 2; BMI 40.0-49.9 kg/m^2^, class 3; BMI >50.0 kg/m^2^, class 4. All values and data were examined retrospectively.

Inclusion and exclusion criteria

The inclusion criteria embraced patients who underwent LSG between August 2023 and August 2024, whose BMI was between 30 and 60 kg/m^2^, and whose age was between 14 and 70 years. Patients who underwent gastric bypass or revisional surgery, who had previous chronic liver disease, or who used daily medication that caused elevated liver function were excluded.

A total of 452 patients were admitted to our center for bariatric surgery. Thirteen were excluded due to revisional surgeries or gastric bypass, one due to chronic liver disease, and two due to the use of medications causing elevated liver function. After the exclusions, 436 patients were approved for inclusion. As this was a retrospective study, ethical approval was not required and informed consent did not apply.

Assessment of hepatic steatosis

Experienced radiologists in our radiology unit performed the USG examinations of all patients included in the study, who were evaluated with the standard approach after eight to 12 hours of fasting preoperatively; the degree of hepatosteatosis and the presence of stones in the gallbladder were recorded. Fatty liver was qualitatively graded into four categories, namely, normal, mild, moderate, and severe (grades 0-3, respectively), based on the following criteria: Grade 0 (normal): The liver parenchyma shows normal echogenicity, and intrahepatic vascular structures and the diaphragm are clearly visualized. Grade 1 (mild): There is a slight increase in parenchymal echogenicity, but intrahepatic vascular structures and the diaphragm remain clearly visible. Grade 2 (moderate): There is a moderate increase in parenchymal echogenicity, with partial visualization of vascular structures and the diaphragm. Grade 3 (severe): The echogenicity is markedly increased, with indistinct or poorly defined borders of vascular structures, the diaphragm, and the posterior lobe [16].

Surgical technique

All patients underwent LSG using the standard five-keyhole technique. First, the greater curvature of the stomach was dissected. After the insertion of a 39 Fr calibration tube, the stomach was separated by staples from 2 to 4 cm away from the pylorus to 1 cm away from the esophagogastric junction. After resection, the entire staple line was imbricated using a V-Loc 2.0 suture. After a leakage test using methylene blue, the operation was completed as previously described [17,18].

Study objectives

The primary outcome of the study was to determine the relationship between the degree of hepatosteatosis and preoperative markers in patients undergoing LSG.

Statical analysis

The mean, median, standard deviation, minimum-maximum, frequency, and ratio were used for descriptive statistics. The Kolmogorov-Smirnov test was used for variable distribution. Quantitative and qualitative independent data were analyzed using the Mann-Whitney U test, independent samples t-test, and chi-squared test. Fischer’s exact test was performed when the conditions were not met. Univariate and multivariate logistic regression was used to investigate the effect level. The IBM SPSS Statistics for Windows, version 28.0 (released 2021, IBM Corp., Armonk, NY) was used for analyses.

## Results

The study included 436 patients, of whom 73.6% (n = 321) were female. The mean age was 37.23 ± 10.49 years, and the mean BMI value was 41.25 ± 6.11 kg/m^2^. The largest group in the population was in obesity class III (n = 199; 45.64%). The most common grades of fatty liver were grades 1 (n = 161; 36.9%) and 2 (n = 156; 35.7%). Among the patients, 27.06% (n = 118) had T2DM, and 9.4% (n = 41) had HT (Table 1). The patients’ biochemical features are shown in Table 2.

**Table 1:** Patient demographics DM: diabetes mellitus, LSG: laparoscopic sleeve gastrectomy, HHR: hiatal hernia repairment, LC: laparoscopic cholecystectomy, sd: standard deviation, min-max: minimum-maximum

Variables	State	Value
Number of patients		436
Gender, n (%)	Female	321 (73.6%)
	Male	115 (26.4%)
Age (years), mean±SD (min–max)		37.23±10.49 (16–67)
Body mass index (kg/m^2^), mean±SD (min–max)		41.25±6.11 (30.1–63.94)
Obesity classification, n (%)	Class I	48 (11.01%)
	Class II	145 (33.26%)
	Class III	199 (45.64%)
	Class IV	44 (10.09%)
Fatty liver disease, n (%)	Grade 0	16 (3.67%)
	Grade 1	161 (36.93%)
	Grade 2	156 (35.78%)
	Grade 3	103 (23.62%)
Presence of gallstone, n (%)	Gallstone (−)	373 (85.55%)
	Gallstone (+)	62 (14.22%)
	Operated	1 (0.23%)
HBsAG, n (%)	(−)	436 (100%)
	(+)	0 (0.0%)
Anti-HCV, n (%)	(−)	436 (100%)
	(+)	0 (0.0%)
Hypertension, n (%)	(−)	395 (90.60%)
	(+)	41 (9.40%)
Type 2 DM, n (%)	(−)	318 (72.94%)
	(+)	118 (27.06%)
Type of surgery, n (%)	LSG	371 (85.1%)
	LSG+HHR	43 (9.86%)
	LSG+LC	20 (4.59%)
	LSG+HHR+LC	2 (0.46%)

**Table 2:** Patient biochemical features AST: aspartate aminotransferase; ALT: alanine aminotransferase; HDL: high-density lipoprotein (mg/dl); LDL: low-density lipoprotein (mg/dl); min–max: minimum–maximum; sd: standard deviation

Variables	Value
AST (U/L), mean±SD (min–max)	28.1 ± 15.7 (9–117)
ALT (U/L), mean±SD (min–max)	33.3 ± 26.0 (4–229)
HDL (mg/dL), mean±SD (min–max)	49.5 ± 13.0 (24–126)
LDL (mg/dL), mean±SD (min–max)	121.7 ± 32.0 (32.1–226)
Triglyceride (mg/dL), mean±SD (min–max)	131.2 ± 92.4 (28–952)
Total cholesterol (mg/dL), mean±SD (min–max)	196.1 ± 39.7 (42–334)
HbA1c (g/dL), mean±SD (min–max)	5.8 ± 1.0 (3.2–14.4)
Fasting blood glucose (mg/dL), mean±SD (min–max)	104.2 ± 32.7 (67–567)
Vitamin D (ng/Ml), mean±SD (min–max)	20.2 ± 14.1 (3.8–138)

When the patients were categorized according to the NAFLD grade, the largest proportion were in grade 1 (n = 161; 37%) and grade 2 (n = 156; 35.6%). The comparison of the groups according to the NAFLD grade revealed no significant difference in age or presence of gallstone, but there were statistically significant differences in weight, BMI, ALT, AST, HDL, LDL, TG, total cholesterol, HbA1c and FBG, vitamin D, obesity class, DM, and HT (p <.05) (Table 3).

**Table 3:** Comparison of risk factors according to the grade of NAFLD M: mean, χ2: chi-square test, ANOVA: analysis of variance, FBG: fasting blood glucose, SGB: stone in the gallbladder, OC: obesity class, TC: total cholesterol, Vit. D: vitamin D, TG: triglyceride, HbA1c: glycated hemoglobin, NoT: name of test

Variables	G0	G1	G2	G3	p-value	NoT
Count, n (%)	16–3.7%	161–36.9%	156–35.8%	103–23.6%	-	-
Age (years) M±SD	38.1 ± 12.1	35.5 ± 9.5	37.8 ± 10.9	38.8 ± 10.8	.06536	F
Weight (kg) M±SD	100.5 ± 14.5	107.3 ± 16.2	116.4 ± 21.0	132.9 ± 24.6	.00000	F
BMI (kg/m^2^) M±SD	36.4 ± 4.4	39.0 ± 4.9	41.42 ± 5.3	45.3 ± 6.9	.00000	F
ALT (U/L) M±SD	19.4 ± 8.6	25.3 ± 14.0	35.04 ± 30.7	45.4 ± 29.3	.00000	F
AST (U/L) M±SD	28.6 ± 11.6	24.8 ± 11.3	27.58 ± 17.3	33.9 ± 18.2	.00008	F
HDL (mg/dL) M±SD	57.4 ± 16.6	52.2 ± 13.6	49.11 ± 13.1	44.59 ± 9.37	.00000	F
LDL (mg/dL) M±SD	112.1 ± 30.0	116.7 ± 28.0	126.09 ± 32.8	124.4 ± 35.9	.02829	F
TG (mg/dL) M±SD	79.3 ± 34.9	114.2 ± 65.4	143.19 ± 98.0	147.9 ± 116.6	.00081	F
TC (mg/dL) M±SD	175.2 ± 48.5	190.5 ± 35.9	203.14 ± 40.6	197.6 ± 40.5	.00489	F
HbA1c (g/dL) M±SD	5.2 ± 0.4	5.5 ± 0.6	5.9 ± 1.1	6.2 ± 1.2	.00000	F
FBG (mg/dL) M±SD	106.9 ± 23.8	97.0 ± 17.0	106.0 ± 25.1	112.4 ± 54.1	.00176	F
Vit.D (ng/ml) M±SD	19.9 ± 8.5	19.3 ± 13.7	22.7 ± 15.9	17.9 ± 11.7	.03682	F
Diabetes, n (%)	0–0.00%	30–18.6%	46–29.5%	42– 40.8%	.00006	χ2
HT, n (%)	1–6.3%	6–3.73%	15–9.6%	19–18.5%	.00105	χ2
SGB, n (%)	0–0.0%	22–13.7%	29–18.6%	12–11.7%	.13285	χ2
OC-1, n (%)	6–37.5%	27–16.8%	11–7.1%	4–3.9%	.0	χ2
OC-2, n (%)	6–37.5%	67–41.6%	50–32.1%	22–21.4%	.008	χ2
OC-3, n (%)	4–25.0%	62–38.5%	86–55.1%	47–45.6%	.008	χ2
OC-4, n (%)	0–0.0%)	5–3.1%	9–5.8%	30–29.1%	.0	χ2

**Figure 1:**
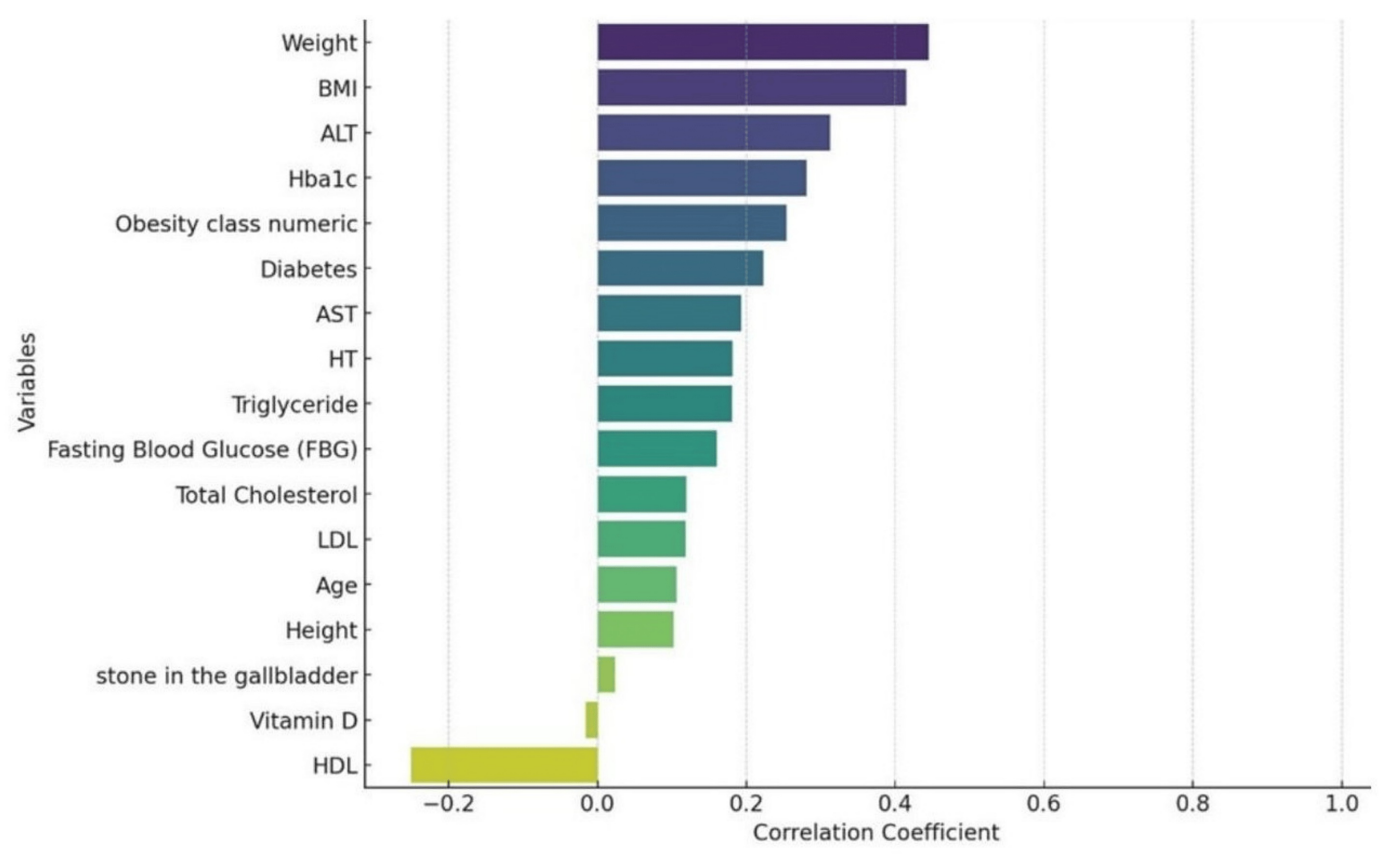
Correlation of variables with hepatic steatosis BMI: body mass index, ALT: alanine aminotransferase, AST: aspartate aminotransferase, HT: hypertension, LDL: low-density lipoprotein, HDL: high-density lipoprotein, HbA1c: glycated hemoglobin

To analyze the factors affecting hepatic steatosis, we performed univariate and multivariate analyses using logistic regression. In the univariate model, there was a strong statistically significant positive association of NAFLD with weight, BMI, ALT, HbA1C, obesity class, AST, TG, total cholesterol, FBG, LDL, and age (p < 0.05). HDL and vitamin D showed a significant inverse relationship with NAFLD. Weight, BMI, and ALT had the highest correlation with hepatosteatosis. In the multivariate analysis, ALT, AST, LDL, total cholesterol, and FBG showed statistically significant associations with NAFLD. As a result of the ROC curve analysis performed to obtain the cut-off value, it was observed that the cut-off value for AST was 22 U/L, the cut-off value for ALT was 29 U/L, the cut-off value for Total cholesterol was 199 mg/dL, the cut-off value for FBG was 100 mg/dL, and the cut-off value for LDL was 178 mg/dL (see Table 4 and Figure 1).

**Table 4:** Univariate and multivariate analyses of factors influencing hepatosteatosis AST: aspartate aminotransferase, ALT: alanine aminotransferase, HDL: high-density lipoprotein (mg/dl), LDL: low-density lipoprotein (mg/dl), HbA1c: glycated hemoglobin

Variables	Univariate model p-value	Multivariate model p-value
Age	0.075924	0.997086
Weight	0.0	0.763826
Body mass index (kg/m^2^)	0.0	0.234713
AST (U/L)	0.000001	0.021002
ALT (U/L)	0.0	0.029838
HDL (mg/dL)	0.000004	0.482345
LDL (mg/dL)	0.029439	0.041194
Triglyceride (mg/dL)	0.000002	0.05853
Total cholesterol (mg/dL)	0.009075	0.039744
HbA1c (g/dL)	0.0	0.054154
Fasting blood glucose (mg/dL)	0.000101	0.004087
Vitamin D (ng/Ml)	0.015135	0.978502
Diabetes mellitus type II	0.000065	0.999719
Hypertension	0.001045	0.347447

## Discussion

Obesity and NAFLD are closely related conditions. An increase in the number of individuals with obesity in society increases the number of newly diagnosed NAFLD patients. NAFLD includes all chronic liver diseases, from simple hepatosteatosis (defined as fat accumulation in the liver without inflammation) to NASH (which is accompanied by inflammation, oxidative stress, cell injury, and fibrosis) [5,6]. Our study evaluated the relationships between fatty liver disease and the risk factors that cause it in patients undergoing LSG.

Although the incidence of fatty liver disease in the population is around 32%, its prevalence varies between 49.1% and 76.3% in the bariatric population when patients are examined before bariatric surgery [6,7,19,20]. In our study, 59.3% of patients were observed to have severe (grade 2-3) fatty liver, whereas only 16 (3.7%) patients did not have a fatty liver. Although the percentage of fatty liver in our study is higher than in the literature, the positive significant relationship between preoperative weight, BMI, and fatty liver in univariate analyses explains the high rate.

Some studies suggest that there is no correlation between BMI and NAFLD. One study found that BMI was an age-dependent risk factor for NAFLD among patients under 35 years of age; patients over 45 years exhibited no relationship between BMI and fatty liver [19,20]. By contrast, some authors suggest that BMI and NAFLD have a statistically significant relationship [6,21]. In our study, there was a statistically strong association between hepatic steatosis and BMI, weight, and obesity class in univariate analyses, but there was no statistically significant relationship in multivariate analyses.

Patients diagnosed with fatty liver disease may have liver enzymes one or two times higher than normal. There are publications showing that elevated ALT is a more effective predictor than BMI in undiagnosed fatty liver diseases, especially due to its close relationship with liver damage. In studies, although a NASH diagnosis gives a statistically significant result in univariate analyses of ALT and AST, multivariate analyses show that only ALT is an independent risk factor for NASH [20,22]. Our study demonstrates that ALT and AST are independent predictors of NAFLD in both uni- and multivariate analyses. ROC curve analysis showed that the cut-off value for ALT was 29 U/L and for AST 22 U/L. ALT had one of the higher correlations with hepatosteatosis.

In the literature, a study showed that NAFLD had a 36.8% association between T2DM and obesity. The same study observed that fatty liver disease was more severe in diabetic patients. Another study found that T2DM was a risk factor but not an independent predictor of NAFLD, whereas HT was both a risk factor and an independent predictor of NAFLD [20,23]. T2DM and FBG have been shown as risk factors, but they were not seen as independent predictive factors for NAFLD [24]. Our study used HbA1c and FBG to determine the severity of T2DM; HbA1c and FBG had strong statistically significant relationships with NAFLD in the analysis of correlation and univariate analysis, but only FBG was an independent risk factor for NAFLD and cut-off value was 100 mg/dL in our study. Moreover, although T2DM and HT were risk factors for NAFLD, we did not observe statistically significant differences in the multivariate analysis.

There are studies in the literature with differing results between dyslipidemia and fatty liver disease. A study that examined risk factors for hepatosteatosis suggests that hypertriglyceridemia is an independent risk factor, with a cut-off value of 140.5 mg/dl for NAFLD. In the same study, there was no correlation between HDL level and hepatosteatosis [24]. Some studies have not found significant relationships between dyslipidemia, diabetes, and NAFLD [20,21]. In our study, there were strong significant relationships between hepatosteatosis and TG, total cholesterol, LDL, and HDL. In the multivariate analysis, total cholesterol and LDL were observed as independent risk factors for NAFLD and we observed that the cut-off value for total cholesterol was 199 mg/dL and for LDL was 178 mg/dL.

There is a known inverse relationship between serum vitamin D levels and fatty liver disease. This can be explained by increased hepatocyte damage caused by the inability of vitamin D at low levels to exert its anti-inflammatory and antifibrotic properties or to increase insulin sensitivity [25,26]. In our study, vitamin D exhibited a significant inverse relationship with the severity of hepatosteatosis.

In sum, our study found that weight, BMI, ALT, AST, HDL, LDL, TG, total cholesterol, FBG, HbA1c, vitamin D, T2DM, and HT were risk factors for NAFLD. Vitamin D and HDL had an inverse correlation with hepatosteatosis. Only ALT, AST, LDL, FBG, and total cholesterol could be used as predictors of NAFLD. When we examined the cut-off value with the ROC curve, we observed that the cut-off value for AST was 22 U/L, for ALT was 29 U/L, for total cholesterol was 199 mg/dL, for FBG was 100 mg/dL, and for LDL was 178 mg/dL. 

Limitations

Our study has some limitations, including its retrospective and single-center nature and a small sample size. We used USG to grade fatty liver disease due to its ease and noninvasive nature, but some studies prefer a liver biopsy and pathological evaluation, which may yield more detailed information and accuracy for grading fatty liver disease. In addition, preoperative biochemical values and diseases related to fatty liver disease were examined in this study. More accurate results could be achieved in a study designed to examine the relationship between the degree of hepatosteatosis and parameters after follow-up time with a comparison of all values.

## Conclusions

Fatty liver disease is a very common condition in patients undergoing bariatric surgery. Our study found that ALT, AST, LDL, FBG, and total cholesterol may serve as predictors of NAFLD, and all these parameters can contribute to determining the severity of hepatosteatosis. More reliable results could be achieved in prospective randomized studies with a larger sample size and longer follow-up.
